# Effect of rhPTH(1-34) and alendronate on the treatment of type 2 diabetic bone disease

**DOI:** 10.3389/fendo.2025.1657481

**Published:** 2025-09-15

**Authors:** Huijuan Li, Lingdan Yuan, Peipei Liu, Yichen Liu, Dongni Huang, Huiru Ding, Wei Jin, Jingnan Liu, Hongxia Wang, Lige Song

**Affiliations:** ^1^ Department of Endocrinology, Yangpu Hospital, School of Medicine, Tongji University, Shanghai, China; ^2^ Department of Endocrinology, Shanghai Tongji Hospital, School of Medicine, Tongji University, Shanghai, China; ^3^ Institute of Osteoporosis and Metabolic Bone Diseases, School of Medicine, Tongji University, Shanghai, China; ^4^ Yichuan Community Health Service Center of Putuo District, Shanghai, China

**Keywords:** diabetic bone disease, rhPTH(1-34), alendronate, bone mineral density, bone turnover

## Abstract

**Introduction:**

Patients with type 2 diabetes mellitus (T2DM) have decreased bone turnover levels. However, there are few studies comparing the anti-osteoporosis effects of anabolic drugs and anti-resorptive drugs in patients with T2DM. Thus, this study was designed to compare the changes in bone mineral density (BMD) and bone turnover levels in mice and postmenopausal osteoporotic patients, both with and without T2DM, following treatment with rhPTH(1-34) or alendronate (ALN).

**Methods:**

In the animal study, the mouse model of T2DM (DM mice) was established by high-fat diet (60% from fat) feeding and streptozotocin injection (100 mg/kg) in C57BL/6 mice. Both DM and control (CON) mice were then randomly assigned to receive either normal saline, rhPTH or ALN treatment. In the clinical study, a single-center, prospective, open-label, randomized controlled clinical trial was conducted. Postmenopausal patients with osteoporosis (OP) and postmenopausal patients with both osteoporosis and type 2 diabetes (DOP) were recruited and randomly assigned to receive either rhPTH(1-34) or ALN treatment for a period of one year. Changes in BMD and bone turnover levels were assessed in all groups.

**Results:**

Compared to CON mice, DM mice exhibited decreased bone mass, impaired bone microstructure and, decreased levels of bone turnover markers, including procollagen type I intact N-terminal (P1NP) and C terminal cross-linking telopeptide of type I collagen (CTX). rhPTH(1-34) could reverse the low bone turnover observed in DM mice and had a better effect on improving BMD, bone volume per tissue volume (BV/TV), trabecular number in femoral trabecular bone, as well as BMD, BV/TV, and trabecular thickness in lumbar trabecular bone. In the clinical trial, at baseline, patients with DOP also exhibited decreased levels of bone turnover markers, including P1NP, CTX and osteocalcin. For patients with DOP, rhPTH(1-34) had a better effect than ALN on BMD improvement at the lumbar spine. Notably, the effect of ALN on lumbar spine improvement in patients with DOP was even smaller than that in patients with OP alone.

**Conclusion:**

Initiating treatment with rhPTH(1-34) may provide greater clinical benefits to patients with diabetic bone disease characterized by low bone turnover levels.

## 1 Introduction

The incidence of fractures in patients with type 2 diabetes mellitus (T2DM) is significantly higher than that in non-diabetic subjects (1). A recent meta-analysis revealed that the relative risks of non-vertebral fractures and hip fractures in patient with T2DM were 1.19 and 1.33 respectively (2). Given the sharp rise in the prevalence of diabetes worldwide (3), the increased fracture risk of patients with T2DM has attracted more attention. Consequently, the term “diabetic bone disease” has been proposed in recent years and is now recognized as one of the chronic complications associated with diabetes.

Previous studies have indicated that patients with T2DM exhibit a reduced level of bone turnover (4). Specifically, the serum levels of procollagen type I intact N-terminal (P1NP) and C terminal cross-linking telopeptide of type I collagen (CTX) were both lower in patients with T2DM (5). However, their femoral and lumbar bone mineral density (BMD) were comparable to or even higher than those of non-diabetic controls (6, 7). Therefore, the anti-osteoporosis treatment strategies for patients with T2DM may need to differ from those applied in non-diabetic patients, considering their unique bone metabolism characteristics.

Bisphosphonate and teriparatide [recombinant human parathyroid hormone 1-34, rhPTH (1–34)] are two widely used anti-osteoporosis medications (8). Bisphosphonates inhibit the bone resorption activity of mature osteoclasts, thereby significantly reducing bone turnover (9). Previous clinical trials have demonstrated that both alendronate (ALN) and risedronate had similar efficacy in suppressing bone turnover and increasing BMD between osteoporosis patients with and without T2DM (10, 11). A large-sample-size cohort study further confirmed that the anti-fracture efficacy of ALN is equivalent in patients with diabetes (12). Conversely, rhPTH (1–34), a potent anabolic agent, stimulates osteoblasts to deposit osteoid, thereby promoting bone formation and increasing bone turnover (13). The DANCE observational study revealed that, compared to non-diabetic patients, rhPTH (1–34) treatment in T2DM patients led to a greater increase in femoral neck BMD, similar increases in spine and total hip BMD and a comparable reduction in nonvertebral fracture incidence (14). However, these results were derived from subgroup analyses or *post-hoc* analyses, primarily focusing on whether there were differences in the effects of anti-osteoporosis drugs between patients with and without diabetes. To date, no randomized clinical trials have directly compared the anti-osteoporosis efficacy of anabolic drugs versus anti-resorptive drugs specifically in patients with T2DM.

Thus, there is still a paucity of evidence regarding the clinical efficacy of anabolic and anti-resorptive drugs in patients with T2DM. This study was designed to compare the changes in BMD and bone turnover levels in mice and postmenopausal osteoporotic patients, both with and without T2DM, following treatment with rhPTH (1–34) or ALN.

## 2 Materials and methods

### 2.1 Animals experiments

#### 2.1.1 Mouse model

The method for establishing the mouse model of type 2 diabetes using high-fat diet (HFD) and streptozotocin (STZ) injection was based on prior researches, with most studies utilizing male mice (15, 16). Male C57BL/6 mice were purchased from Vital River (Zhejiang, China) and housed under specific pathogen-free condition. Four-week-old mice were randomly divided into the control group (CON) or the type 2 diabetes group (DM). After one week of adaptive feeding, mice were fed with HFD (TP23500, 60% kcal from fat, Trophic Animal Feed, China) for 9 weeks. Mice in the DM group were then intraperitoneally injected with STZ (Sigma-Aldrich) dissolved in citrate buffer (0.1 mol/L, pH=4.5). The dose of STZ was 100 mg/kg body weight. Mice in the CON group were intraperitoneally injected with citrate buffer alone. On days 7, 14, and 21 after STZ injection, fasting blood glucose (after an 8-hour fast) was measured. If the value was ≥16.7 mmol/L on all three occasions, the mice were considered to have successfully developed diabetes. Oral glucose tolerance test (OGTT, 1.5 g/kg glucose by gavage) and insulin tolerance test (ITT, 0.75 IU/kg insulin by intraperitoneal injection) were conducted to evaluate glucose metabolism, with a one-week interval between the two tests, prior to sacrifice. Blood glucose levels were measured by a glucometer (ACCU-CHECK^®^ Performa, Roche). At 17 weeks of age, the CON mice and the DM mice were randomly divided into the following three group respectively (1): normal saline group (2); rhPTH group (3); ALN group. Each experimental group had 5 mice.

At 17 weeks of age, alendronate (A4978, Sigma-Aldrich) were administrated to mice in ALN group at a dose of 2 mg/kg per week by gavage for 11 weeks, based on previous studies (17, 18). At 20 weeks of age, teriparatide (rhPTH, Forsteo, Eli Lilly) were administrated to mice in rhPTH group at a dose of 80 μg/kg per day by subcutaneous injection for 8 weeks, based on previous studies (19, 20). All mice were sacrificed at 28 weeks of age.

Animals were maintained in facilities operated by Shanghai Tongji Hospital, School of Medicine, Tongji University, and all animal experimental procedures were approved by the Institutional Animal Care and Use Committee of the Shanghai Tongji Hospital (2021-DW-020).

#### 2.1.2 Microcomputed tomography (micro-CT) analysis in mice

Micro-CT analysis has been described in our previous study (17). Briefly, the femurs and the third lumbar vertebrae (L3) were fixed in 4% paraformaldehyde for 36 hours, and then scanned by a micro-CT scanner (SkyScan 1176, Bruker, Kontich, Belguim) using 8.96 μm voxel size, 45 KV, 500 μA and 0.6 degrees rotation step (180 degrees angular range) and analyzed by CT Analyser (Version: 1.15.4.0+, Bruker, Kontich, Belguim). For trabecular bone analysis in the femur, a 1 mm region of metaphyseal spongiosa was evaluated in the distal femur, positioned 0.5 mm proximal to the growth plate. Trabecular bone in the lumbar vertebra was quantified within a 1 mm region positioned 0.7 mm proximal to the growth plate. For cortical bone analysis, a 0.5 mm segment at the femoral mid-diaphysis (50% of bone length) was measured. The grayscale quantification thresholds were set at 85–255 for trabecular bone and 115–255 for cortical bone. The volumetric bone mineral density (BMD, g/cm^3^), trabecular bone volume per tissue volume (BV/TV, %), trabecular thickness (Tb.Th., mm), trabecular space (Tb.Sp., mm), trabecular number (Tb.N., 1/mm), cortical thickness (Ct.Th., mm) and cortical total porosity (Ct. Porosity, %) were assessed.

#### 2.1.3 Histological analysis

The femurs and the third lumbar vertebrae (L3) were fixed in 4% paraformaldehyde for 36 hours and decalcified with 20% ethylenediaminetetraacetic acid (EDTA). Then specimens were embedded in paraffin and sectioned at four micrometers according to standard histological procedures. Hematoxylin-eosin (HE) staining and tartrate-resistant acid phosphatase (TRACP) staining was performed using staining kits (Beyotime and Solarbio respectively).

#### 2.1.4 Von Kossa staining and *in vivo* mineral apposition rate measurement

The femurs were fixed in 4% paraformaldehyde for 48 hours. Following fixation, the tissues were rehydrated and cleared. Finally, the tissues were embedded in a mixture of methyl methacrylate, dibutyl phthalate, and benzoyl peroxide. Ten-micrometer sections were used to perform Von Kossa staining using staining kits (Macklin). For *in vivo* mineral apposition rate measurement, mice were intraperitoneally injected with calcein (20 mg/kg body weight, Sigma-Aldrich) on days 11 and 2 prior to sacrifice. Images were captured using a fluorescence microscope (Nikon, DS-Ri2). Mineral apposition rate (MAR, μm/d) was calculated from the distance between two fluorochrome double labels.

#### 2.1.5 Biochemical parameters measurement

Blood samples were collected from 28-week-old mice after 6-hour fasting. Serum triglyceride (mmol/L) and serum total cholesterol (mmol/L) were detected by a commercial kit from Nanjing Jiancheng. Serum insulin (nIU/mL) were detected using enzyme-linked immunosorbent assay (ELISA) kits from CUSABIO (Cusabio Biotech Cat# CSB-E05071m, RRID: AB_2916335, coefficient of variation of intra- and inter-assay <10%). Serum P1NP (ng/mL) and serum CTX (ng/mL) were measured using ELISA kits from Immunodiagnostic Systems Limited (P1NP: Immunodiagnostic Systems Cat# AC-33F1, RRID: AB_2801263; CTX: Immunodiagnostic Systems Cat# AC-06F1, RRID: AB_2801265; coefficient of variation of intra- and inter-assay <15%).

### 2.2 Clinical trial

#### 2.2.1 Study population

In accordance with the Guidelines for the Diagnosis and Treatment of Primary Osteoporosis issued by the Chinese Society of Osteoporosis and Bone Mineral Research, which stipulate that the use of rhPTH(1-34) is restricted to postmenopausal osteoporotic patients, we recruited two groups of participants: postmenopausal patients with osteoporosis but without type 2 diabetes mellitus (OP), and postmenopausal patients with osteoporosis and type 2 diabetes mellitus (DOP). Patients were all collected from the Department of Endocrinology, Tongji Hospital, School of Medicine, Tongji University, Shanghai, China. The calculation of sample size was based on the mean percentage change in lumbar spine BMD. According to a previous study, the mean percentage increase in lumbar spine BMD from baseline to week 48 (approximately 12 months) was 1.3 ± 5.0(%) in the alendronate group and 5.9 ± 5.5(%) in the rhPTH(1-34) group (21). Therefore, a sample size of 38 is required to achieve a statistical power of 80% at a significance level of 5%.

The inclusion criteria were as follows: 1) ambulatory postmenopausal women aged between 65 to 80 years (inclusive); 2) all subjects met the osteoporosis diagnostic criteria issued by the International Osteoporosis Foundation (IOF) guideline (2019), and patients with type 2 diabetes met the diagnostic criteria for type 2 diabetes issued by the World Health Organization (WHO) guideline (1999); 3) have history of lumbar vertebral fragility fracture in the past one year; 4) general condition is good and do not need to rely on others for help in daily life; 5) no previous use of any anti-osteoporosis drugs; 6) willing to sign informed consent and to adhere to the study protocol. The exclusion criteria were as follows: 1) have diseases or drugs that cause secondary osteoporosis such as rheumatoid arthritis, hyperparathyroidism, hyperthyroidism, glucocorticoid, etc.; 2) thiazolidinediones were used within 1 year before screening or during follow-up; 3) renal dysfunction (estimated glomerular filtration rate <60 mL/min/1.73m^2^); 4) serum 25-hydroxyvitamin D [25(OH)D] concentration <50 nmol/L; 5) malignant tumor; 6) current enrollment in other drug trials or less than 1 month since completion; 7) contraindications to teriparatide or bisphosphonate therapy.

This study was approved by the Ethics Committee of Tongji Hospital, Tongji University School of Medicine (2022-043) and complied with the ethical standards of the Declaration of Helsinki. All the patients signed written informed consent.

#### 2.2.2 Study design

This clinical trial was a single-center, prospective, open-label, randomized controlled study conducted in Tongji Hospital, School of Medicine, Tongji University, Shanghai, China. Randomization was performed using a computer-generated sequence (SPSS) by an independent statistician. Allocation concealment was ensured using sequentially numbered, opaque, sealed envelope, which was opened only after baseline assessments. Outcome assessors and data analysts were blinded to treatment allocation throughout the study period. A per-protocol analysis was performed to handle dropouts. Subjects with OP and subjects with DOP were respectively randomized 1:1 to receive teriparatide (rhPTH; Forsteo, Eli Lilly, 20 ug per day, subcutaneously) or alendronate (ALN; Fosamax, Merck, 70 mg per week, orally). Throughout the study period, all participants were administered a daily regimen of 525 IU of vitamin D3 and 600 mg of calcium supplementation.

The primary endpoint was the percentage change in areal bone mineral density (aBMD) at the lumbar spine from baseline to month 12. The secondary endpoints included the percentage changes in aBMD at the femoral neck and total hip from baseline to month 12, and the percentage changes in serum bone alkaline phosphatase (BALP), serum P1NP, serum osteocalcin (OC) and serum CTX from baseline to month 6 and month 12.

#### 2.2.3 Data collection

Age, menopausal time and history of fracture were recorded. aBMD (g/cm^2^) at lumbar spine 1-4, femoral neck and total hip were detected by dual-energy X-ray absorptiometry (DXA, HOLOGIC Discovery; coefficient of variation <1%). aBMD was detected at baseline and month 12. Peripheral venous blood was collected after overnight fasting. Serum P1NP (ng/mL), OC (ng/mL) and CTX (ng/mL) were measured by electrochemiluminescence assay (P1NP: Roche Cat# 03141071190, RRID: AB_2782967; OC: Roche Cat# 12149133122, RRID: AB_2915903; CTX: Roche Cat# 11972308122, RRID: AB_2905599; coefficient of variation of intra- and inter-assay <10%). Serum BALP (μg/L) was measured by enzyme immunoassay (Immunodiagnostic Systems Cat# AC-20F1, RRID: AB_2943063, coefficient of variation of intra- and inter-assay <10%). Serum bone turnover markers were all measured at baseline, month 6 and month 12.

### 2.3 Statistical analysis

Statistical analysis was conducted using SPSS Statistics version 22.0 and GraphPad Prism version 8.0. Continuous variables were expressed as mean ± standard deviation (SD). Comparison between two groups was conducted using the Student’s t-test or the Mann-Whitney U test, depending on whether the data were normally distributed. For more than two groups, one-way analysis of variance (one-way ANOVA) with LSD test was applied. Categorical variables were reported as numbers and percentages, with Fisher’s exact test used for comparison. *p*<0.05 was considered to be statistically significant.

## 3 Results

### 3.1 Mice with DM exhibited impaired glucose tolerance and insulin resistance

**Figure 1:**
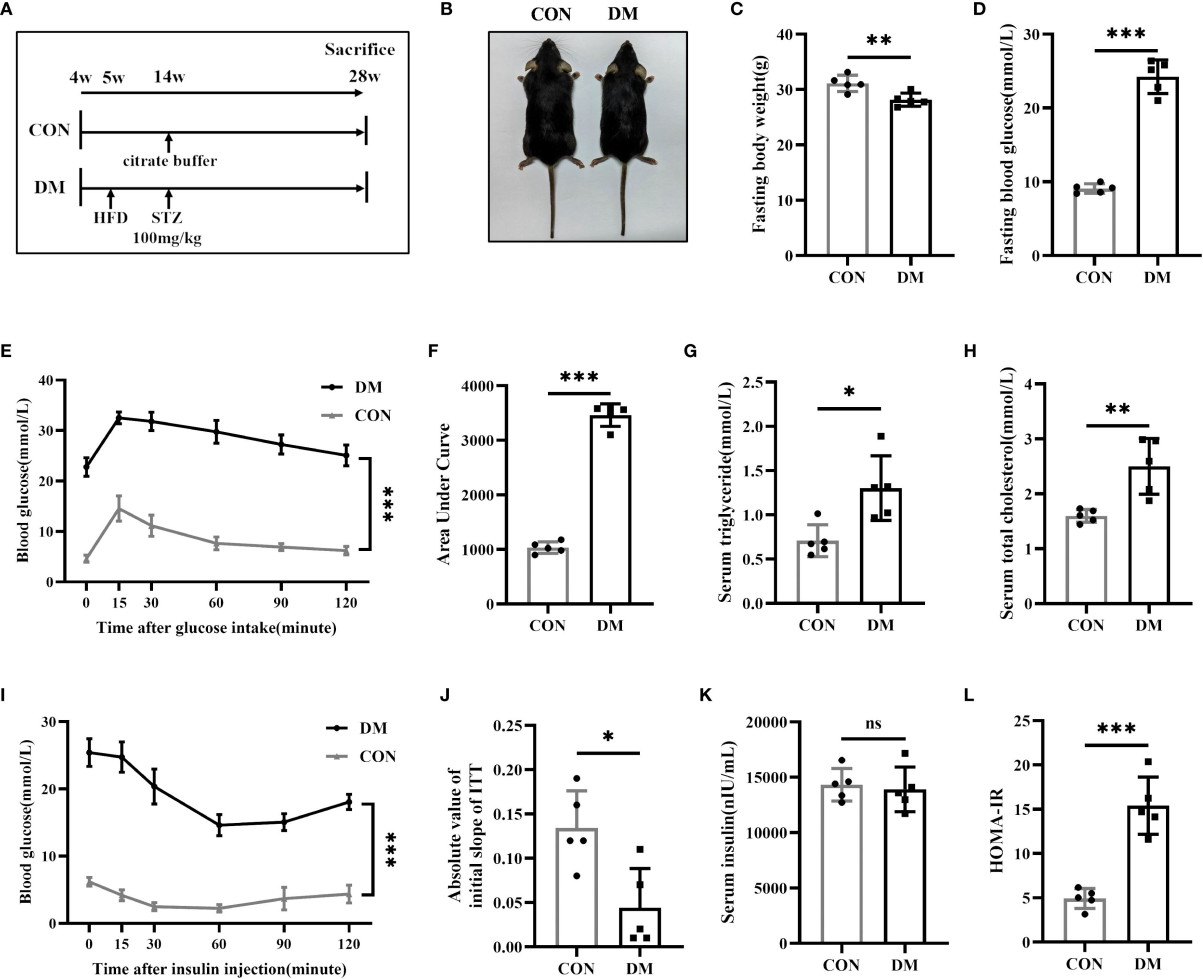
Establishment of type 2 diabetes mice. **(A)** Experimental protocol. Four-week-old mice were divided into the CON group or DM group (n = 5 per group). After 9 weeks of high-fat diet (HFD) feeding, mice in DM group were intraperitoneally injected with streptozotocin (STZ, 100 mg/kg). All mice were sacrificed at 28-week-old of age. **(B)** Gross images of CON mice and DM mice at 28-week-old of age. **(C, D)** Fasting body weight and fasting blood glucose of CON and DM mice at 28-week-old of age (n = 5 per group). **(E, F)** Blood glucose levels after gavage of glucose (1.5 g/kg). The areas under the curves indicated glucose tolerance (n = 5 per group). **(G, H)** Levels of serum triglyceride and serum total cholesterol (n = 5 per group). **(I)** Blood glucose levels after an intraperitoneal insulin injection (0.75 IU/kg) (n = 5 per group). **(J)** Absolute value of slope of insulin tolerance test (ITT) during 0-15minute (n = 5 per group). **(K, L)** Levels of serum insulin and HOMA-IR index (n = 5 per group). CON, control mice; DM, type 2 diabetes mice. HOMA-IR=fasting blood glucose(mmol/L)*fasting insulin(μIU/mL)/22.5. Data are represented as means ± SD. *p<0.05, **p<0.01, ***p<0.001, DM mice compared to CON mice.

The mouse model of diabetic bone disease was established in C57BL/6 mice through a combination of high-fat diet feeding and STZ injection (**Figure 1A**). Gross images of CON mice and DM mice are shown in **Figure 1B**. At 28 weeks of age, the body weight of DM mice was slightly lower than that of CON mice, while blood glucose levels were significantly higher (**Figures 1C, D**). OGTT revealed impaired glucose tolerance in DM mice (**Figures 1E, F**). Additionally, DM mice showed impaired lipid metabolism, with elevated serum triglyceride (**Figure 1G**) and total cholesterol levels (**Figure 1H**). Although serum insulin levels did not differ significantly between the two groups (**Figure 1K**), DM mice exhibited a reduced absolute value of the initial slope (during 0–15 minute) of the ITT curve (**Figures 1I, J**) and an increased HOMA-IR level (**Figure 1L**), indicating the presence of insulin resistance in DM mice.

### 3.2 Mice with DM exhibited reduced bone mass, compromised bone microstructure, and diminished bone turnover

**Figure 2:**
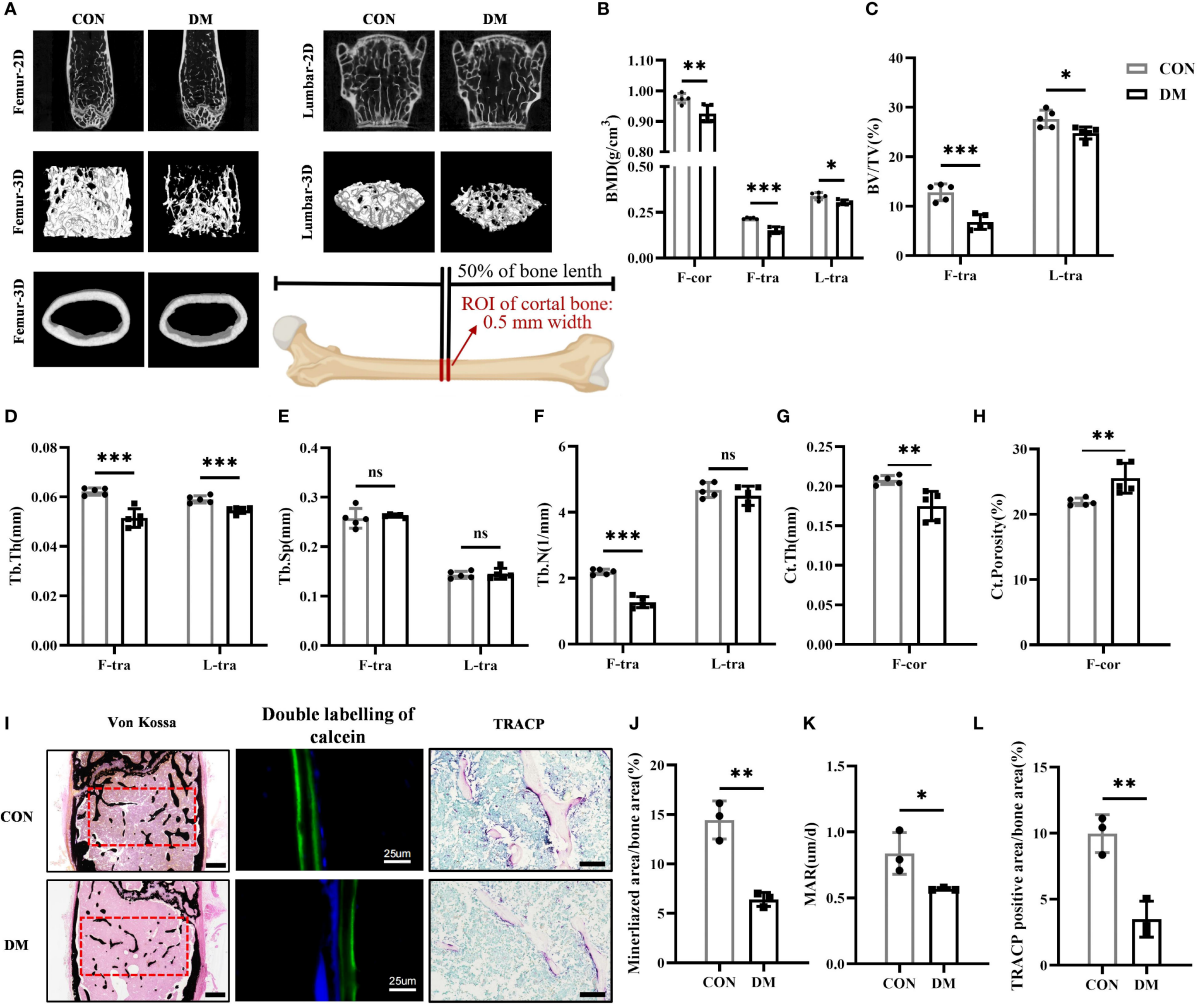
DM mice had impaired bone structure and lower bone turnover level. **(A)** Representative micro-CT 2D and 3D images of the femur and L3 vertebra at 28-week-old of age (n = 5 per group) and a schematic diagram illustrating the region of interest (ROI) for cortical bone analysis. **(B)** Volumetric bone mineral density (BMD) quantification of femoral cortical bone (F-cor), femoral trabecular bone (F-tra), and L3 vertebral trabecular bone (L-tra) (n = 5 per group). **(C–F)** Trabecular bone parameters in the femur and L3 vertebra including bone volume per tissue volume (BV/TV, %), trabecular thickness (Tb.Th., mm), trabecular space (Tb.Sp., mm) and trabecular number (Tb.N., 1/mm) (n = 5 per group). **(G, H)** Cortical bone parameters including cortical thickness (Ct.Th., mm) and total porosity (Ct. Porosity, %) in the femur (n = 5 per group). **(I)** Von Kossa staining (scale bar, 400 μm), double labeling of calcein (scale bar, 25 μm) and tartrate-resistant acid phosphatase (TRACP) staining (scale bar, 100 μm) of femur (n = 3 per group). **(J)** Percentage of mineralized bone area calculated by Image J program (n = 3 per group). **(K)** Analysis of mineral apposition rate (MAR) in 28-week-old CON and DM mice (n = 3 per group). **(L)** Percentage of TRACP positive area calculated by Image J program (n = 3 per group). CON, control mice; DM, type 2 diabetes mice. Data are represented as means ± SD. *p<0.05, **p<0.01, ***p<0.001, DM mice compared to CON mice.

Micro-CT analysis revealed that DM mice exhibited significantly reduced BMD and BV/TV in both femurs and lumbar vertebrae compared to CON mice (**Figures 2A–C**). Additionally, DM mice showed lower Tb.Th and Tb.N in femoral trabecular bone, as well as decreased Tb.Th in lumbar trabecular bone (**Figures 2D, F**). However, no statistically significant differences in Th.Sp were observed between DM and CON mice in either femurs or lumbar vertebrae (**Figure 2E**). Ct.Th of femurs was also decreased in DM mice, and Ct.Porosity was increased (**Figures 2G, H**). Therefore, DM mice had experienced a substantial decline in bone mass and compromised bone microarchitecture. Von Kossa staining (**Figures 2I, J**), double labeling of calcein fluorescence (**Figures 2I, K**), and TRACP staining (**Figures 2I, L**) collectively demonstrated that the mineralized bone tissue volume, mineral apposition rate, and bone resorption activity were all significantly reduced in DM mice compared to controls, suggesting a decreased level of bone turnover in DM mice.

### 3.3 Both rhPTH and ALN were capable of improving bone mass and bone microstructure in DM mice, but rhPTH demonstrated better efficacy

**Figure 3:**
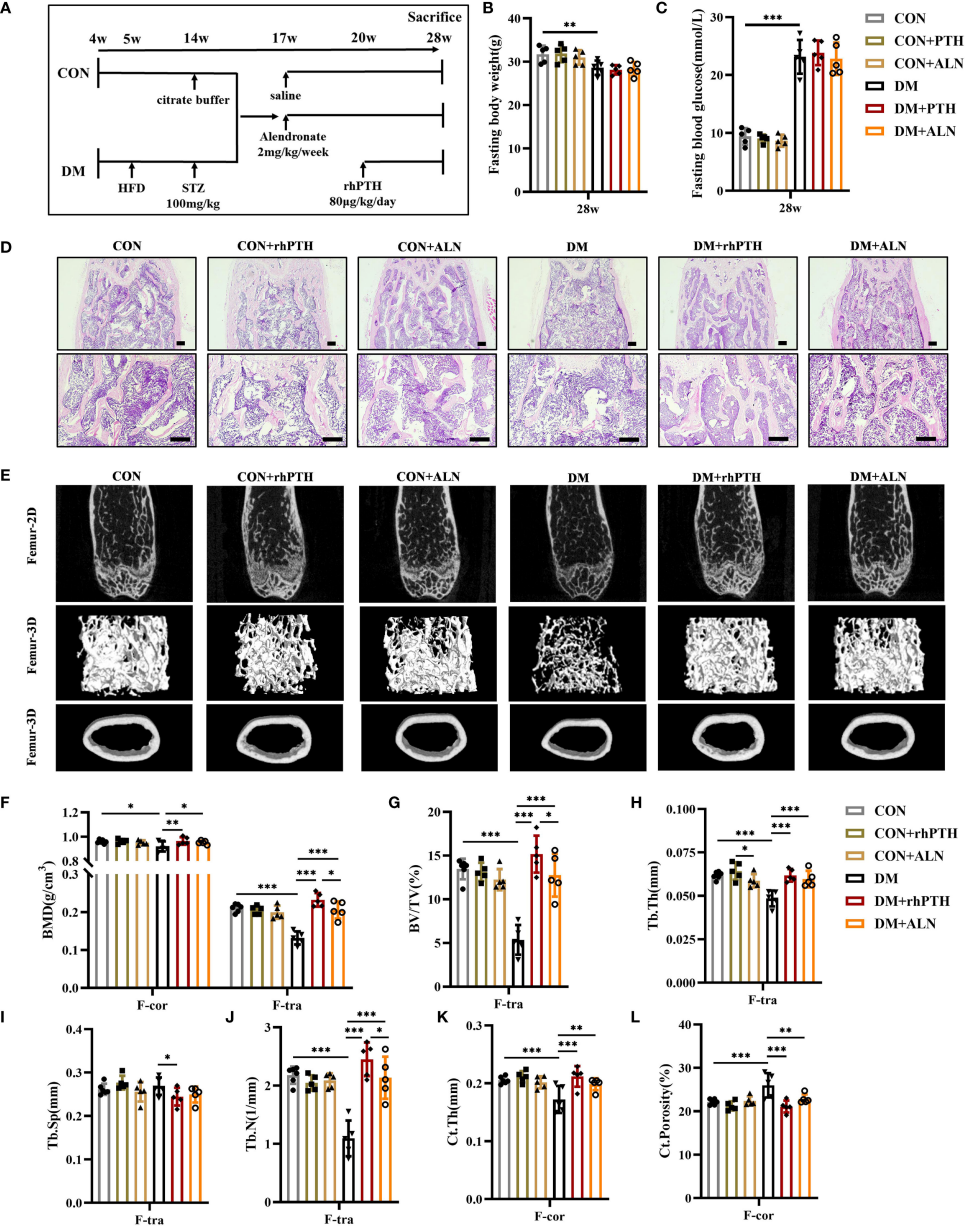
Decreased femoral bone mass in DM mice could be rescued by rhPTH or ALN treatment, but rhPTH was more effective. **(A)** Experimental protocol. CON and DM mice were respectively assigned to three different groups (n = 5 per group). All mice were sacrificed at 28-week-old of age. **(B, C)** Fasting body weight and fasting blood glucose of mice at 28-week-old of age (n = 5 per group). **(D)** Representative images of HE staining of femur at 28-week-old of age (n = 5 per group). Scale bar, 200 μm. **(E)** Representative micro-CT 2D and 3D images of the femur at 28-week-old of age (n = 5 per group). **(F)** Volumetric bone mineral density (BMD) quantification of femoral cortical bone (F-cor) and femoral trabecular bone (F-tra) (n = 5 per group). **(G–J)** Femoral trabecular bone parameters including bone volume per tissue volume (BV/TV, %), trabecular thickness (Tb.Th., mm), trabecular space (Tb.Sp., mm) and trabecular number (Tb.N., 1/mm) (n = 5 per group). **(K, L)** Cortical bone parameters including cortical thickness (Ct.Th., mm) and total porosity (Ct. Porosity, %) in the femur (n = 5 per group). CON, control mice; DM, type 2 diabetes mice; rhPTH, recombinant human parathyroid hormone; ALN, alendronate. Data are represented as means ± SD. *p<0.05, **p<0.01, ***p<0.001.

Mice were treated with rhPTH or ALN to compare the therapeutic effects of these two drugs (**Figure 3A**). Neither rhPTH nor ALN administration affected the body weight and blood glucose levels of the mice (**Figures 3B, C**). According to the HE staining and micro-CT images, both rhPTH and ALN effectively enhanced bone mass in the femurs of mice with DM (**Figures 3D, E**). In DM mice, the BMD and BV/TV of femoral trabecular bone, as well as the BMD of femoral cortical bone, were significantly increased following treatment with either rhPTH or ALN (**Figures 3F, G**). However, rhPTH exhibited a more pronounced therapeutic effect on improving BMD and BV/TV in femoral trabecular bone (**Figures 3F, G**). Additionally, rhPTH was more effective than ALN in increasing femoral Tb.N in DM mice and was able to reduce femoral Tb.Sp, whereas ALN did not have the capability to improve femoral Tb.Sp (**Figures 3I, J**). Regarding the impaired Tb.Th, Ct.Th, and Ct.Porosity of femurs in DM mice, both rhPTH and ALN showed similar efficacy (**Figures 3H, K–L**).

**Figure 4:**
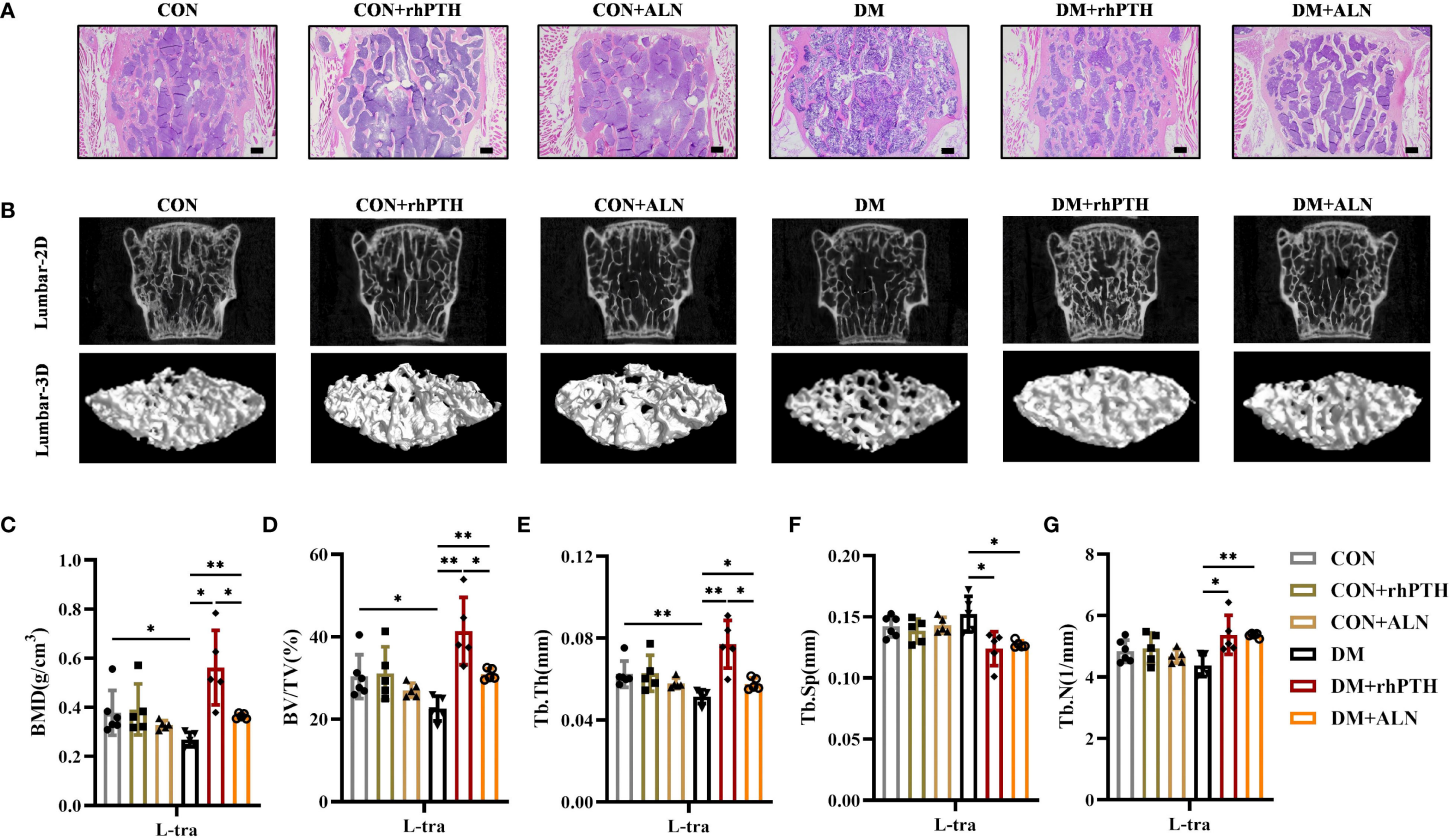
Decreased lumbar bone mass in DM mice could be rescued by rhPTH or ALN treatment, but rhPTH was more effective. **(A)** Representative images of HE staining of L3 vertebra at 28-week-old of age (n = 5 per group). Scale bar, 200 μm. **(B)** Representative micro-CT 2D and 3D images of the L3 vertebra at 28-week-old of age (n = 5 per group). **(C)** Volumetric bone mineral density (BMD) quantification of L3 vertebral trabecular bone (L-tra) (n = 5 per group). **(D–G)** Lumbar vertebral trabecular bone parameters including bone volume per tissue volume (BV/TV, %), trabecular thickness (Tb.Th., mm), trabecular space (Tb.Sp., mm) and trabecular number (Tb.N., 1/mm) (n = 5 per group). CON, control mice; DM, type 2 diabetes mice; rhPTH, recombinant human parathyroid hormone; ALN, alendronate. Data are represented as means ± SD. *p<0.05, **p<0.01.

Similar to their effects on femurs, both rhPTH and ALN were able to improve the low bone mass of lumbar vertebrae in DM mice (**Figures 4A, B**). However, rhPTH was more effective in increasing BMD, BV/TV, and Tb.Th in lumbar vertebrae (**Figures 4C–E**). Regarding lumbar Tb.Sp and Tb.N, there was no significant difference in the effectiveness between rhPTH and ALN (**Figures 4F, G**).

### 3.4 rhPTH significantly elevated bone turnover in DM mice

**Figure 5:**
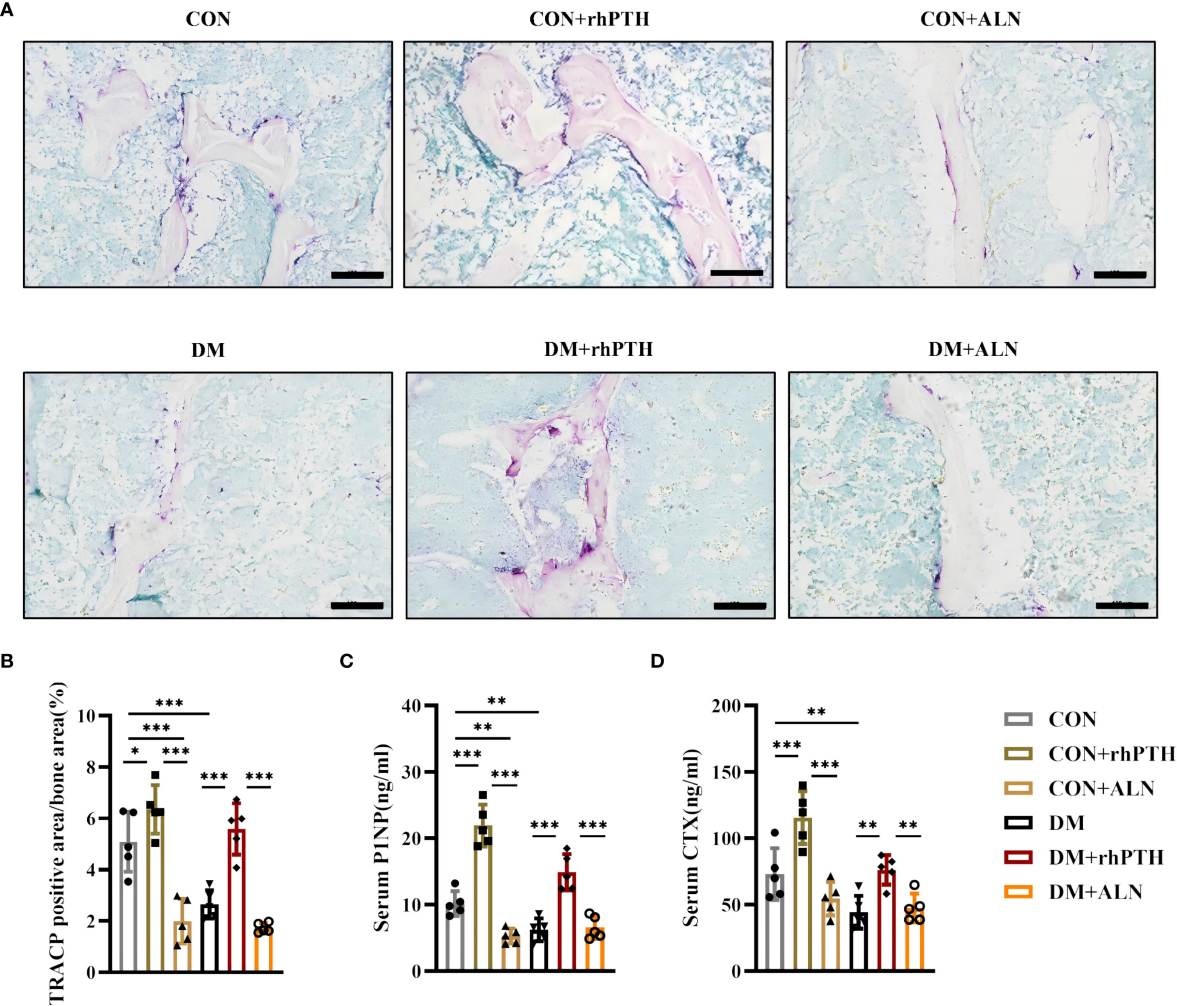
rhPTH had a better effect on improving bone turnover level in DM mice. **(A)** Representative images of tartrate-resistant acid phosphatase (TRACP) staining of femur (n = 5 per group). Scale bar, 100 μm. **(B)** Percentage of TRACP positive area calculated by Image J program (n = 5 per group). **(C, D)** Serum procollagen type I intact N-terminal (P1NP) and C-terminal cross-linking telopeptide of type I collagen (CTX) concentrations determined by ELISA (n = 5 per group). CON, control mice; DM, type 2 diabetes mice; rhPTH, recombinant human parathyroid hormone; ALN, alendronate. Data are represented as means ± SD. *p<0.05, **p<0.01, ***p<0.001.

As depicted in **Figure 2**, mice with DM exhibited reduced levels of both bone formation and bone resorption. To further investigate the impact of rhPTH and ALN on bone turnover, we performed TRACP staining on femurs and measured the serum levels of the bone formation marker P1NP and the bone resorption marker CTX. rhPTH significantly elevated the percentage of TRACP-positive area in the femurs of mice with DM (**Figures 5A, B**) and increased serum levels of P1NP and CTX (**Figures 5C, D**). Conversely, following ALN treatment, mice with DM showed a decreasing trend in TRACP-positive area in the femurs (**Figures 5A, B**), while serum P1NP and ,,CTX levels remained low (**Figures 5C, D**).

### 3.5 Compared to ALN, rhPTH more effectively improved lumbar spine bone mineral density in patients with DOP

**Figure 6:**
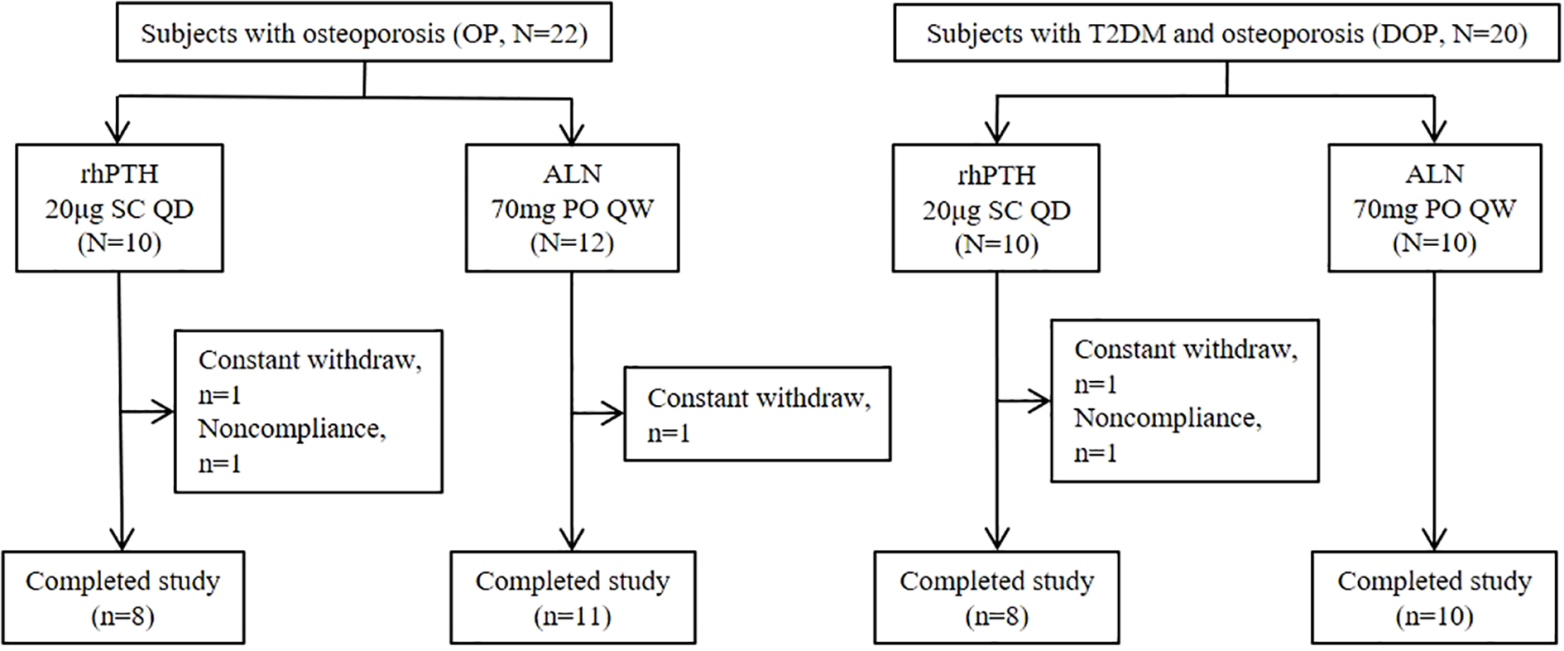
Subject disposition. rhPTH, recombinant human parathyroid hormone; ALN, alendronate; QD, every day; QW, every week.

In mice with DM, rhPTH effectively reversed the low bone turnover and demonstrated superior efficacy compared to ALN in improving bone mass and bone microstructure. To further validate the aforementioned findings in human subjects, we recruited two groups of participants: postmenopausal patients with osteoporosis but without type 2 diabetes mellitus (OP), and postmenopausal patients with osteoporosis and type 2 diabetes mellitus (DOP). Data were collected from May 2022 to December 2023. A total of 42 patients were enrolled in this study, with 37 patients (88.1%) completing the follow-up (**Figure 6**). Based on the primary endpoint, which is the percentage change in lumbar spine aBMD, the power of this study is 99%. All patients had a history of lumbar vertebral fragility fractures within one year prior to enrollment according to the inclusion criteria. At baseline, patients with DOP showed higher levels of serum BALP and lower levels of serum P1NP, OC and CTX, indicating a low bone turnover status similar to that observed in mice with T2DM (**Table 1**). Moreover, no significant differences were detected in the history of fracture, aBMD of the lumbar spine, femoral neck, and total hip between OP and DOP patients (**Table 1**). Patients with OP and DOP were then randomized to receive either rhPTH or ALN treatment for a 12-month period. Notably, baseline characteristics including age, years since menopause, fracture history, bone turnover markers, and aBMD were comparable between the rhPTH and ALN subgroups, with no statistically significant differences observed (**Table 1**).

**Table 1:** Baseline demographics and bone metabolism characteristics.

Factors	OP(n=19)	DOP(n=18)	OP		DOP		*p* value		
			OP+rhPTH (n=8)	OP+ALN (n=11)	DOP+rhPTH (n=8)	DOP+ALN (n=10)	OP vs DOP	OP+rhPTH vs OP+ALN	DOP+rhPTH vs DOP+ALN
Age (years)	67.37 ± 7.97	67.61 ± 6.69	68.00 ± 7.58	66.91 ± 8.58	68.63 ± 7.21	66.80 ± 6.51	0.921	0.778	0.581
Years since menopause (years)	17.89 ± 6.65	17.78 ± 6.49	17.63 ± 7.58	18.09 ± 6.27	18.50 ± 7.09	17.20 ± 6.29	0.957	0.885	0.686
History of fracture (%)									
Lumbar vertebral fracture	19 (100%)	18 (100%)	8 (100%)	11 (100%)	8 (100%)	10 (100%)	/	/	/
Hip fracture	1 (5.26%)	1 (5.56%)	0 (0%)	1 (9.09%)	0 (0%)	1 (10.00%)	1.000	1.000	1.000
Wrist fracture	6 (31.58%)	5 (27.78%)	2 (25.00%)	4 (36.36%)	3 (37.50%)	2 (20.00%)	1.000	1.000	0.608
Proximal humerus fracture	2 (10.53%)	3 (16.67%)	1 (12.50%)	1 (9.09%)	1 (12.50%)	2 (20.00%)	0.660	1.000	1.000
Baseline BTM levels									
BALP (μg/L)	15.77 ± 2.46	19.47 ± 4.76	15.92 ± 2.78	15.66 ± 2.34	20.17 ± 5.12	18.92 ± 4.64	0.016*	0.702	0.696
P1NP (ng/mL)	54.19 ± 9.74	43.17 ± 8.79	53.59 ± 11.38	54.63 ± 8.92	45.33 ± 9.51	41.44 ± 8.26	0.001**	0.825	0.367
OC (ng/mL)	21.12 ± 4.20	14.49 ± 2.64	21.70 ± 4.15	20.70 ± 4.39	14.58 ± 2.61	14.42 ± 2.80	<0.001***	0.623	0.903
CTX (ng/mL)	0.55 ± 0.13	0.38 ± 0.13	0.54 ± 0.14	0.56 ± 0.13	0.40 ± 0.13	0.38 ± 0.13	<0.001***	0.732	0.660
Baseline aBMD levels									
Lumbar spine (g/cm^2^)	0.72 ± 0.06	0.71 ± 0.05	0.71 ± 0.05	0.72 ± 0.07	0.71 ± 0.04	0.71 ± 0.06	0.878	0.967	0.930
Femoral neck (g/cm^2^)	0.54 ± 0.08	0.54 ± 0.07	0.53 ± 0.07	0.55 ± 0.08	0.53 ± 0.08	0.55 ± 0.06	0.828	0.657	0.372
Total hip (g/cm^2^)	0.70 ± 0.09	0.69 ± 0.07	0.69 ± 0.08	0.70 ± 0.09	0.70 ± 0.07	0.69 ± 0.07	0.658	0.762	0.914

OP, patients with osteoporosis; DOP, patients with osteoporosis and type 2 diabetes; rhPTH, recombinant human parathyroid hormone; ALN, alendronate; BALP, bone alkaline phosphatase; P1NP, procollagen type I intact N-terminal; OC, osteocalcin; CTX, C-terminal cross-linking telopeptide of type I collagen; BTM,bone turnover marker; aBMD, areal bone mineral density. Data are represented as means ± SD. *p<0.05, **p<0.01, ***p<0.001.

**Figure 7:**
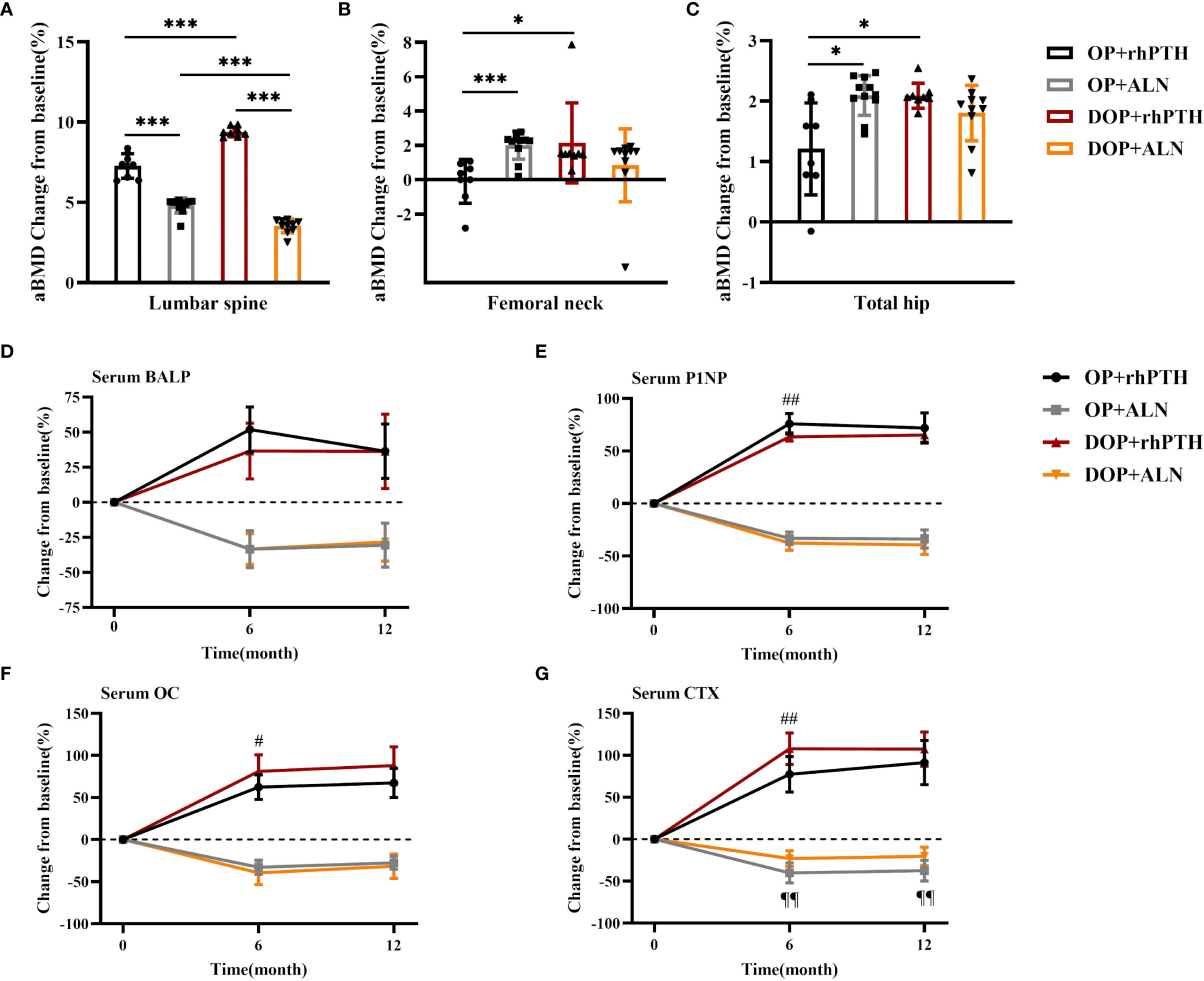
rhPTH had a better effect on improving lumbar bone density in DOP patients. **(A–C)** Mean percentage change (%) of aBMD after treatment (n = 8–11 per group). * *p*<0.05, *** *p*<0.001. **(D–G)** Mean percentage change (%) of serum bone turnover marker concentrations after treatment (n = 8–11 per group). # *p*<0.05, ## *p*<0.01, DOP+rhPTH compared to OP+rhPTH. ¶¶ *p*<0.01, DOP+ALN compared to OP+ALN. OP, patients with osteoporosis; DOP, patients with type 2 diabetes and osteoporosis; rhPTH, recombinant human parathyroid hormone; ALN, alendronate; aBMD, areal bone mineral density; BALP, bone alkaline phosphatase; P1NP, procollagen type I intact N-terminal; OC, osteocalcin; CTX, C-terminal cross-linking telopeptide of type I collagen. Data are represented as means ± SD.

The percentage changes in aBMD at the lumbar spine, femoral neck and total hip were calculated from baseline to month 12. For the lumbar spine, rhPTH showed a better effect on aBMD improvement compared to ALN in both OP patients [(7.27 ± 0.77 vs 4.80 ± 0.47)%, *p*<0.001] and DOP patients [(9.38 ± 0.31 vs 3.54 ± 0.43)%, *p*<0.001] (**Table 2**, **Figure 7A**). The percentage increase in lumbar spine aBMD was higher in the DOP+rhPTH group than in the OP+rhPTH group [(9.38 ± 0.31 vs 7.27 ± 0.77)%, *p*<0.001] (**Table 2**, **Figure 7A**). Conversely, the percentage increase in lumbar spine aBMD was lower in the DOP+ALN group compared to the OP+ALN group [(3.54 ± 0.43 vs 4.80 ± 0.47)%, *p*<0.001] (**Table 2**, **Figure 7A**). In the femoral neck and total hip, rhPTH and ALN showed similar effects on improving aBMD in DOP patients [femoral neck: (2.15 ± 2.33 vs 0.84 ± 2.13)%, *p* = 0.986; total hip: (2.09 ± 0.21 vs 1.80 ± 0.46)%, *p* = 0.101] (**Table 2**, **Figures 7B, C**). However, in OP patients, rhPTH was less effective than ALN in enhancing aBMD at the femoral neck [(-0.10 ± 1.28 vs 2.00 ± 0.81)%, *p*<0.001] (**Table 2**, **Figure 7B**) and total hip [(1.21 ± 0.76 vs 2.09 ± 0.33)%, *p* = 0.013] (**Table 2**, **Figure 7C**). Notably, the improvement in aBMD at the femoral neck and total hip was greater in the DOP+rhPTH group than in the OP+rhPTH group [femoral neck: (2.15 ± 2.33 vs -0.10 ± 1.28)%, *p* = 0.026; total hip: (2.09 ± 0.21 vs 1.21 ± 0.76)%, *p* = 0.013] (**Table 2**, **Figures 7B, C**).

**Table 2:** Percentage changes in aBMD from baseline to month 12.

Factors	OP+rhPTH (n=8)	OP+ALN (n=11)	DOP+rhPTH (n=8)	DOP+ALN (n=10)	*p* value OP+rhPTH vs OP+ALN	*p* value OP+rhPTH vs DOP+rhPTH	*p* value DOP+rhPTH vs DOP+ALN	*p* value OP+ALN vs DOP+ALN
Change of aBMD levels (12 months)								
Lumbar spine(%)	7.27 ± 0.77	4.80 ± 0.47	9.38 ± 0.31	3.54 ± 0.43	<0.001***	<0.001***	<0.001***	<0.001***
Femoral neck(%)	-0.10 ± 1.28	2.00 ± 0.81	2.15 ± 2.33	0.84 ± 2.13	<0.001***	0.026*	0.986	0.076
Total hip(%)	1.21 ± 0.76	2.09 ± 0.33	2.09 ± 0.21	1.80 ± 0.46	0.013*	0.013*	0.101	0.118

OP, patients with osteoporosis; DOP, patients with osteoporosis and type 2 diabetes; rhPTH, recombinant human parathyroid hormone; ALN, alendronate; aBMD, areal bone mineral density. Data are represented as means ± SD. *p<0.05, **p<0.01, ***p<0.001.

Regarding bone turnover markers, after 6 months of treatment, the increase in serum P1NP was greater in the OP+rhPTH group than in the DOP+rhPTH group [(75.97 ± 9.95 vs 63.34 ± 4.15)%, *p* = 0.005] (**Table 3**, **Figure 7E**). Conversely, the increases in serum OC and CTX were higher in the DOP+rhPTH group compared to the OP+rhPTH group [OC: (81.16 ± 19.68 vs 62.30 ± 14.62)%, *p* = 0.047; CTX: (107.79 ± 18.85 vs 77.43 ± 21.00)%, *p* = 0.009] (**Table 3**, **Figures 7F, G**). After 12 months of treatment, there were no statistically significant differences in the changes of serum bone turnover markers between the OP+rhPTH and DOP+rhPTH groups (**Table 3**, **Figures 7D–G**). As for the effects of ALN on OP and DOP patients, the percentage decrease in serum CTX was greater in the OP+ALN group compared to the DOP+ALN group after 6 months of treatment [(-40.18 ± 11.87 vs -23.00 ± 9.47)%, *p* = 0.002] (**Table 3**, **Figure 7G**), and this difference persisted throughout the 12-month treatment period [(-37.44 ± 12.27 vs -20.30 ± 10.80)%, *p* = 0.003] (**Table 3**, **Figure 7G**).

**Table 3:** Percentage changes in bone turnover markers from baseline to month 6 and month 12.

Factors	OP+rhPTH(n=8)	DOP+rhPTH(n=8)	*p* value	OP+ALN(n=11)	DOP+ALN(n=10)	*p* value
Change of BTM levels (6 months)						
BALP (%)	51.88 ± 16.05	36.53 ± 19.95	0.112	-33.44 ± 13.17	-33.35 ± 11.08	0.985
P1NP (%)	75.97 ± 9.95	63.34 ± 4.15	0.005**	-33.12 ± 6.11	-37.56 ± 6.82	0.132
OC (%)	62.30 ± 14.62	81.16 ± 19.68	0.047*	-32.77 ± 8.51	-39.52 ± 13.95	0.192
CTX (%)	77.43 ± 21.00	107.79 ± 18.85	0.009**	-40.18 ± 11.87	-23.00 ± 9.47	0.002**
Change of BTM levels (12 months)						
BALP (%)	36.40 ± 19.45	36.35 ± 26.56	0.997	-30.57 ± 15.67	-28.28 ± 13.56	0.725
P1NP (%)	71.87 ± 14.34	65.31 ± 6.92	0.263	-33.82 ± 8.69	-39.34 ± 9.09	0.171
OC (%)	67.33 ± 17.32	87.98 ± 22.26	0.057	-27.63 ± 7.49	-31.61 ± 14.34	0.429
CTX (%)	91.32 ± 26.20	107.38 ± 20.49	0.194	-37.44 ± 12.27	-20.30 ± 10.80	0.003**

OP, patients with osteoporosis; DOP, patients with osteoporosis and type 2 diabetes; rhPTH, recombinant human parathyroid hormone; ALN, alendronate; BALP, bone alkaline phosphatase; P1NP, procollagen type I intact N-terminal; OC, osteocalcin; CTX, C-terminal cross-linking telopeptide of type I collagen; BTM, bone turnover marker. Data are represented as means ± SD. *p<0.05, **p<0.01, ***p<0.001.

## 4 Discussion

### 4.1 Decreased bone turnover in T2DM

Many studies have revealed abnormal bone metabolism in diabetic subjects. Leptin receptor-deficiency (db/db) type 2 diabetic mice model, despite having increased body weight which is benefit for bone mass, had decreased bone mass in both tibia and the fifth lumbar vertebral body although the magnitude of the decrease in the vertebral body was less than that of tibia (22). T2DM mice and rats induced by high-fat diet and STZ injection also exhibited a decrease in femoral BV/TV, trabecular number, trabecular thickness and cortical BMD (16, 23–26), which is similar to the results in this paper. In contrast to the diabetic mice model, no differences in aBMD were found between patients with DOP and OP, consistent with earlier studies showing that patients with type 2 diabetes had equal or even higher bone mineral density than non-diabetic controls (6, 7). However, they are still at a higher risk of fracture. The alterations in bone microarchitecture and composition which can finally result in compromised bone quality may explain the paradoxical phenomenon of retained BMD and increased bone fragility (27). A large, community-based study revealed that older T2DM patients had decreased cortical volumetric bone mineral density, decreased cross-sectional area and increased cortical porosity at the tibia, measured by HR-pQCT (28). Another study with large sample size also revealed lower cross-sectional area and smaller cortical area of tibia in older male T2DM patients (29). Trabecular bone score (TBS), a parameter evaluating lumbar spine microarchitecture and negatively correlated to fracture risk, was lower in T2DM patients according to a meta-analysis (30). Altered bone composition was also found in trabecular bone of T2DM patients, leading to lower energy absorption (31). In addition, decreased bone turnover can also lead to reduced bone strength and increased fracture risk (32). T2DM mice and rats appeared to have reduced bone formation and bone resorption, evidenced by decreased mineral apposition rate (33, 34), decreased serum concentrations of bone turnover markers including P1NP (25, 35), osteocalcin (36) and CTX (25, 37), consistent to our results. Patients with T2DM also showed decreased levels of serum P1NP, osteocalcin, CTX and TRACP (5, 38, 39). Histomorphometric analysis also revealed decreased bone formation rate and decreased osteoblast surface in T2DM patients (40).

### 4.2 Mechanisms underlying decreased bone turnover in T2DM

All the abnormalities that occur in T2DM subjects can affect bone turnover by impairing the function of osteoblasts or osteoclasts. High glucose disrupted the osteogenic potential of bone marrow mesenchymal stem cells (BMSCs) (41) and inhibited the proliferation and differentiation of osteoblasts through inducing pyroptosis (42). Receptor activator of nuclear factor−κB ligand (RANKL)−induced osteoclast differentiation was also inhibited by high glucose through downregulation of key molecules including v −ATPase V0 subunit d2 and dendritic cell−specific transmembrane protein (43). In addition to high glucose, the accumulation of advanced glycation end products (AGE) could also suppress the differentiation and function of osteoclasts and osteoblasts, thereby induced low bone turnover (44). Furthermore, an increased bone marrow fat fraction has been observed in both patients with T2DM and corresponding animal models (45). According to the results from a co-culture system, free fatty acids released by the adipocytes inhibited osteoblasts proliferation, reduced the expression levels of runt-related transcription factor 2, type I collagen and osteocalcin, and induced osteoblasts apoptosis by activating ROS-ERK/P38 signaling pathway (46). Another study revealed that high free fatty acids and high glucose activated METTL3/ASK1-p38 signaling pathway and induced osteoblast ferroptosis (23). Increased oxidative stress and chronic inflammatory status also played an important role in diabetic bone disease. Activation of ROS/MAPKs/NF-κB/NLRP3 pathway inhibited the efferocytosis of osteoclasts (47). Chronic inflammation stimulated NF-κB activities in osteoblasts and suppressed osteoblast function (48).

### 4.3 Therapeutic implications of targeting low bone turnover in diabetic osteoporosis

Theoretically, anabolic therapies may be more effective in T2DM subjects characterized by a low-bone-turnover state. An animal experiment showed that rhPTH(1-34) seemed to have better effect than risedronate on improving vertebral BMD of SDT fatty rats, a rat model of T2DM, but they did not compare the effect directly (49). A retrospective cohort study showed that rhPTH(1-34) could increase the BMD of the lumbar spine, whole femur and femoral neck in type 2 diabetic patients with osteoporosis, and the change in BMD of the femoral neck in the rhPTH(1-34) group tended to be higher than that in the bisphosphonates groups, although the difference was not statistically significant (50). Furthermore, an integrated analysis of four prospective observational studies showed a greater reduction in the rate of clinical fractures with rhPTH(1-34) treatment among diabetic patients compared to non-diabetic patients, although the type of diabetes was not clearly classified (51). As for the effect of bisphosphonate on diabetic patients, a retrospective study showed that elderly, obese, osteoporotic, postmenopausal women with T2DM were resistant to long-term bisphosphonates use (4.8 years), especially in the hip, femoral neck, and forearm (52). To date, no large-scale randomized controlled clinical trials have been conducted to compare the therapeutic effects of anabolic agents and anti-resorptive agents in osteoporotic patients with T2DM. The findings of this study indicated that rhPTH(1-34) could reverse the low bone turnover observed in mice with T2DM, and indeed had a better effect on improving BMD, BV/TV, trabecular number in femoral trabecular bone, as well as BMD, BV/TV, trabecular thickness in lumbar trabecular bone. As for osteoporotic patients with T2DM, our results revealed that rhPTH(1-34) had a better effect than ALN on aBMD improvement at the lumbar spine. Notably, the effect of ALN on lumbar spine improvement in osteoporotic patients with T2DM was even smaller than that in patients with osteoporosis alone. The results above suggest that rhPTH(1-34) may be a more beneficial treatment option for patients with T2DM to treat osteoporosis. Additionally, it is interesting to note that the CON mice showed a lack of response to rhPTH, potentially due to their physiologically balanced bone turnover and relatively high bone volume. As demonstrated by recent findings, mice with high baseline BV/TV exhibit a diminished anabolic response to exogenous PTH (53). The homeostatic balance of baseline bone turnover, near-peak physiological bone formation capacity, and receptor saturation by endogenous PTH may collectively attenuate the osteogenic effect of exogenous PTH stimulation.

### 4.4 Study limitations and translational relevance

This study still has some limitations. Specifically, the T2DM mouse model utilized in this study did not fully replicate the normal aBMD observed in patients with T2DM. Moreover, the clinical trial was conducted as a single-center, small sample size, and open-label study, which may have influenced the results. However, this study further confirms the low bone turnover status in both mice and patients with T2DM and this is the first study to compare the efficacy of rhPTH(1-34) and ALN in the treatment of diabetic bone disease in T2DM. Despite utilizing male mice in the animal experiments and enrolling postmenopausal women in the clinical trial, the concordant low bone turnover status observed in both subjects ensures the significant clinical relevance of this study’s findings.

## 5 Conclusion

In conclusion, compared to ALN, rhPTH(1-34) is more effective in reversing low bone mass, impaired bone microstructure, and low bone turnover levels in mice with T2DM. Additionally, rhPTH(1-34) had a better effect than ALN on improving lumbar spine aBMD in osteoporotic patients with T2DM. These findings suggest that initiating treatment with rhPTH(1-34) may provide greater clinical benefits to patients with diabetic bone disease characterized by low bone turnover levels. However, further confirmation through multicenter, prospective, randomized controlled clinical studies is necessary to validate these results and to comprehensively compare the fracture prevention effects of these two drugs.

## Data Availability

The original contributions presented in the study are included in the article/supplementary material. Further inquiries can be directed to the corresponding author.

## References

[1] Jia P Bao L Chen H Yuan J Liu W Feng F. Risk of low-energy fracture in type 2 diabetes patients: a meta-analysis of observational studies. Osteoporosis Int. (2017) 28:3113–21. doi: 10.1007/s00198-017-4183-0, PMID: 28795239

[2] Vilaca T Schini M Harnan S Sutton A Poku E Allen IE. The risk of hip and non-vertebral fractures in type 1 and type 2 diabetes: A systematic review and meta-analysis update. Bone. (2020) 137:115457. doi: 10.1016/j.bone.2020.115457, PMID: 32480023

[3] Sun H Saeedi P Karuranga S Pinkepank M Ogurtsova K Duncan BB. IDF Diabetes Atlas: Global, regional and country-level diabetes prevalence estimates for 2021 and projections for 2045. Diabetes Res Clin Pract. (2022) 183:109119. doi: 10.1016/j.diabres.2021.109119, PMID: 34879977 PMC11057359

[4] Wu B Fu Z Wang X Zhou P Yang Q Jiang Y. A narrative review of diabetic bone disease: Characteristics, pathogenesis, and treatment. Front Endocrinol (Lausanne). (2022) 13:1052592. doi: 10.3389/fendo.2022.1052592, PMID: 36589835 PMC9794857

[5] Hygum K Starup-Linde J Harsløf T Vestergaard P Langdahl BL. Mechanisms in endocrinology: Diabetes mellitus, a state of low bone turnover - a systematic review and meta-analysis. Eur J Endocrinol. (2017) 176:R137–r57. doi: 10.1530/eje-16-0652, PMID: 28049653

[6] Mostafa SM Elebrashy I Haddad HE Shaker O Razek NA Fayed A. Association between bone turnover markers, bone mineral density, and serum osteoglycine in middle-aged men with Type 2 Diabetes mellitus. Diabetol Metab Syndrome. (2024) 16:155. doi: 10.1186/s13098-024-01388-8, PMID: 38982537 PMC11232153

[7] Li H Wen Y Liu P Zhang L Zhang X Liu Y. Characteristics of bone metabolism in postmenopausal women with newly diagnosed type 2 diabetes mellitus. Clin Endocrinol (Oxf). (2021) 95:430–8. doi: 10.1111/cen.14501, PMID: 34008210

[8] Mohsin S Baniyas MM AlDarmaki RS Tekes K Kalász H Adeghate EA. An update on therapies for the treatment of diabetes-induced osteoporosis. Expert Opin Biol Ther. (2019) 19:937–48. doi: 10.1080/14712598.2019.1618266, PMID: 31079501

[9] Barbosa JS Almeida Paz FA Braga SS. Bisphosphonates, old friends of bones and new trends in clinics. J Med Chem. (2021) 64:1260–82. doi: 10.1021/acs.jmedchem.0c01292, PMID: 33522236

[10] Keegan TH Schwartz AV Bauer DC Sellmeyer DE Kelsey JL. Effect of alendronate on bone mineral density and biochemical markers of bone turnover in type 2 diabetic women: the fracture intervention trial. Diabetes Care. (2004) 27:1547–53. doi: 10.2337/diacare.27.7.1547, PMID: 15220226

[11] Inoue D Muraoka R Okazaki R Nishizawa Y Sugimoto T. Efficacy and safety of risedronate in osteoporosis subjects with comorbid diabetes, hypertension, and/or dyslipidemia: A *post hoc* analysis of phase III trials conducted in Japan. Calcified Tissue Int. (2016) 98:114–22. doi: 10.1007/s00223-015-0071-9, PMID: 26466937 PMC4723633

[12] Vestergaard P Rejnmark L Mosekilde L. Are antiresorptive drugs effective against fractures in patients with diabetes? Calcified Tissue Int. (2011) 88:209–14. doi: 10.1007/s00223-010-9450-4, PMID: 21161194

[13] Neer RM Arnaud CD Zanchetta JR Prince R Gaich GA Reginster JY. Effect of parathyroid hormone (1-34) on fractures and bone mineral density in postmenopausal women with osteoporosis. New Engl J Med. (2001) 344:1434–41. doi: 10.1056/nejm200105103441904, PMID: 11346808

[14] Schwartz AV Pavo I Alam J Disch DP Schuster D Harris JM. Teriparatide in patients with osteoporosis and type 2 diabetes. Bone. (2016) 91:152–8. doi: 10.1016/j.bone.2016.06.017, PMID: 27374026

[15] Luo J Quan J Tsai J Hobensack CK Sullivan C Hector R. Nongenetic mouse models of non-insulin-dependent diabetes mellitus. Metabol: Clin Experimen. (1998) 47:663–8. doi: 10.1016/s0026-0495(98)90027-0, PMID: 9627363

[16] Chen X Yang K Sun P Zhao R Liu B Lu P. Exercise improves bone formation by upregulating the Wnt3a/β-catenin signalling pathway in type 2 diabetic mice. Diabetol Metab Syndrome. (2021) 13:116. doi: 10.1186/s13098-021-00732-6, PMID: 34688315 PMC8542289

[17] Song L Li H Liu Y Zhang X Wen Y Zhang K. Postnatal deletion of β-catenin in preosteoblasts regulates global energy metabolism through increasing bone resorption and adipose tissue fibrosis. Bone. (2022) 156:116320. doi: 10.1016/j.bone.2021.116320, PMID: 34973494

[18] Coe LM Tekalur SA Shu Y Baumann MJ McCabe LR. Bisphosphonate treatment of type I diabetic mice prevents early bone loss but accentuates suppression of bone formation. J Cell Physiol. (2015) 230:1944–53. doi: 10.1002/jcp.24929, PMID: 25641511 PMC4724196

[19] Le Henaff C Ricarte F Finnie B He Z Johnson J Warshaw J. Abaloparatide at the same dose has the same effects on bone as PTH (1-34) in mice. J Bone Mminer Res. (2020) 35:714–24. doi: 10.1002/jbmr.3930, PMID: 31793033 PMC7145759

[20] Yu C Xuan M Zhang M Yao Q Zhang K Zhang X. Postnatal deletion of β-catenin in osterix-expressing cells is necessary for bone growth and intermittent PTH-induced bone gain. J Bone Miner Metab. (2017) 36:560–72. doi: 10.1007/s00774-017-0873-0, PMID: 29124436

[21] Kobayashi N Inaba Y Uchiyama M Ike H Kubota S Saito T. Teriparatide versus alendronate for the preservation of bone mineral density after total hip arthroplasty - A randomized controlled trial. J Arthroplasty. (2016) 31:333–8. doi: 10.1016/j.arth.2015.07.017, PMID: 26260784

[22] Williams GA Callon KE Watson M Costa JL Ding Y Dickinson M. Skeletal phenotype of the leptin receptor–deficient db/db mouse. J Bone Mminer Res. (2011) 26:1698–709. doi: 10.1002/jbmr.367, PMID: 21328476

[23] Lin Y Shen X Ke Y Lan C Chen X Liang B. Activation of osteoblast ferroptosis via the METTL3/ASK1-p38 signaling pathway in high glucose and high fat (HGHF)-induced diabetic bone loss. FASEB J. (2022) 36:e22147. doi: 10.1096/fj.202101610R, PMID: 35104016

[24] Yang Y Lin Y Wang M Yuan K Wang Q Mu P. Targeting ferroptosis suppresses osteocyte glucolipotoxicity and alleviates diabetic osteoporosis. Bone Res. (2022) 10:26. doi: 10.1038/s41413-022-00198-w, PMID: 35260560 PMC8904790

[25] Huang KC Chuang PY Yang TY Tsai YH Li YY Chang SF. Diabetic rats induced using a high-fat diet and low-dose streptozotocin treatment exhibit gut microbiota dysbiosis and osteoporotic bone pathologies. Nutrients. (2024) 16:1220. doi: 10.3390/nu16081220, PMID: 38674910 PMC11054352

[26] Xian Y Liu B Shen T Yang L Peng R Shen H. Enhanced SIRT3 expression restores mitochondrial quality control mechanism to reverse osteogenic impairment in type 2 diabetes mellitus. Bone Res. (2025) 13:30. doi: 10.1038/s41413-024-00399-5, PMID: 40025004 PMC11873136

[27] Li GF Zhao PP Xiao WJ Karasik D Xu YJ Zheng HF. The paradox of bone mineral density and fracture risk in type 2 diabetes. Endocrine. (2024) 85:1100–3. doi: 10.1007/s12020-024-03926-w, PMID: 38922479

[28] Samelson EJ Demissie S Cupples LA Zhang X Xu H Liu CT. Diabetes and deficits in cortical bone density, microarchitecture, and bone size: framingham HR-pQCT study. J Bone Mminer Res. (2018) 33:54–62. doi: 10.1002/jbmr.3240, PMID: 28929525 PMC5771832

[29] Faraj M Schwartz AV Burghardt AJ Black D Orwoll E Strotmeyer ES. Risk factors for bone microarchitecture impairments in older men with type 2 diabetes - the mrOS study. J Clin Endocrinol Metab. (2024) 110:e1660–9. doi: 10.1210/clinem/dgae452, PMID: 38994585 PMC12012815

[30] Ho-Pham LT Nguyen TV. Association between trabecular bone score and type 2 diabetes: a quantitative update of evidence. Osteoporosis Int. (2019) 30:2079–85. doi: 10.1007/s00198-019-05053-z, PMID: 31214749

[31] Sihota P Yadav RN Dhaliwal R Bose JC Dhiman V Neradi D. Investigation of mechanical, material, and compositional determinants of human trabecular bone quality in type 2 diabetes. J Clin Endocrinol Metab. (2021) 106:e2271–e89. doi: 10.1210/clinem/dgab027, PMID: 33475711

[32] Sheu A Greenfield JR White CP Center JR. Contributors to impaired bone health in type 2 diabetes. Trends Endocrinol Metabol: TEM. (2023) 34:34–48. doi: 10.1016/j.tem.2022.11.003, PMID: 36435679

[33] Xu CY Xu C Xu YN Du SQ Dai ZH Jin SQ. Poliumoside protects against type 2 diabetes-related osteoporosis by suppressing ferroptosis via activation of the Nrf2/GPX4 pathway. Phytomedicine. (2024) 125:155342. doi: 10.1016/j.phymed.2024.155342, PMID: 38295665

[34] Jin C Tan K Yao Z Lin BH Zhang DP Chen WK. A novel anti-osteoporosis mechanism of VK2: interfering with ferroptosis via AMPK/SIRT1 pathway in type 2 diabetic osteoporosis. J Agric Food Chem. (2023) 71:2745–61. doi: 10.1021/acs.jafc.2c05632, PMID: 36719855

[35] Behera J Ison J Voor MJ Tyagi N. Exercise-linked skeletal irisin ameliorates diabetes-associated osteoporosis by inhibiting the oxidative damage-dependent miR-150-FNDC5/pyroptosis axis. Diabetes. (2022) 71:2777–92. doi: 10.2337/db21-0573, PMID: 35802043 PMC9750954

[36] Shi P Hou A Li C Wu X Jia S Cen H. Continuous subcutaneous insulin infusion ameliorates bone structures and mechanical properties in type 2 diabetic rats by regulating bone remodeling. Bone. (2021) 153:116101. doi: 10.1016/j.bone.2021.116101, PMID: 34245934

[37] Aeimlapa R Wongdee K Tiyasatkulkovit W Kengkoom K Krishnamra N Charoenphandhu N. Anomalous bone changes in ovariectomized type 2 diabetic rats: inappropriately low bone turnover with bone loss in an estrogen-deficient condition. Am J Physiol Endocrinol Metab. (2019) 317:E646–e57. doi: 10.1152/ajpendo.00093.2019, PMID: 31361547

[38] Liu XX Jiang L Liu Q Zhang J Niu W Liu J. Low bone turnover markers in young and middle-aged male patients with type 2 diabetes mellitus. J Diabetes Res. (2020) 2020:6191468. doi: 10.1155/2020/6191468, PMID: 32851096 PMC7436354

[39] Hunt HB Miller NA Hemmerling KJ Koga M Lopez KA Taylor EA. Bone tissue composition in postmenopausal women varies with glycemic control from normal glucose tolerance to type 2 diabetes mellitus. J Bone Mminer Res. (2021) 36:334–46. doi: 10.1002/jbmr.4186, PMID: 32970898

[40] Manavalan JS Cremers S Dempster DW Zhou H Dworakowski E Kode A. Circulating osteogenic precursor cells in type 2 diabetes mellitus. J Clin Endocrinol Metab. (2012) 97:3240–50. doi: 10.1210/jc.2012-1546, PMID: 22740707 PMC3431571

[41] Cao B Liu N Wang W. High glucose prevents osteogenic differentiation of mesenchymal stem cells via lncRNA AK028326/CXCL13 pathway. Biomed Pharmacother. (2016) 84:544–51. doi: 10.1016/j.biopha.2016.09.058, PMID: 27693963

[42] Yang L Liu J Shan Q Geng G Shao P. High glucose inhibits proliferation and differentiation of osteoblast in alveolar bone by inducing pyroptosis. Biochem Biophys Res Commun. (2020) 522:471–8. doi: 10.1016/j.bbrc.2019.11.080, PMID: 31780258

[43] Xu J Yue F Wang J Chen L Qi W. High glucose inhibits receptor activator of nuclear factor−κB ligand-induced osteoclast differentiation via downregulation of v−ATPase V0 subunit d2 and dendritic cell−specific transmembrane protein. Mol Med Rep. (2015) 11:865–70. doi: 10.3892/mmr.2014.2807, PMID: 25352342 PMC4262508

[44] Park SY Choi KH Jun JE Chung HY. Effects of advanced glycation end products on differentiation and function of osteoblasts and osteoclasts. J Korean Med Sci. (2021) 36:e239. doi: 10.3346/jkms.2021.36.e239, PMID: 34581519 PMC8476938

[45] Kim TY Schafer AL. Diabetes and bone marrow adiposity. Curr Osteoporosis Rep. (2016) 14:337–44. doi: 10.1007/s11914-016-0336-x, PMID: 27714580 PMC5126977

[46] Dong X Bi L He S Meng G Wei B Jia S. FFAs-ROS-ERK/P38 pathway plays a key role in adipocyte lipotoxicity on osteoblasts in co-culture. Biochimie. (2014) 101:123–31. doi: 10.1016/j.biochi.2014.01.002, PMID: 24424405

[47] An Y Zhang H Wang C Jiao F Xu H Wang X. Activation of ROS/MAPKs/NF-κB/NLRP3 and inhibition of efferocytosis in osteoclast-mediated diabetic osteoporosis. FASEB J. (2019) 33:12515–27. doi: 10.1096/fj.201802805RR, PMID: 31461386 PMC6902677

[48] Chang J Wang Z Tang E Fan Z McCauley L Franceschi R. Inhibition of osteoblastic bone formation by nuclear factor-kappaB. Nat Med. (2009) 15:682–9. doi: 10.1038/nm.1954, PMID: 19448637 PMC2768554

[49] Nomura S Kitami A Takao-Kawabata R Takakura A Nakatsugawa M Kono R. Teriparatide improves bone and lipid metabolism in a male rat model of type 2 diabetes mellitus. Endocrinology. (2019) 160:2339–52. doi: 10.1210/en.2019-00239, PMID: 31504411 PMC6760306

[50] Munekawa C Hashimoto Y Kitagawa N Osaka T Hamaguchi M Fukui M. Effect of teriparatide on bone mineral density and trabecular bone score in type 2 diabetic patients with osteoporosis: A retrospective cohort study. Med (Kaunas Lithuania). (2022) 58:481. doi: 10.3390/medicina58040481, PMID: 35454320 PMC9030978

[51] Langdahl BL Silverman S Fujiwara S Saag K Napoli N Soen S. Real-world effectiveness of teriparatide on fracture reduction in patients with osteoporosis and comorbidities or risk factors for fractures: Integrated analysis of 4 prospective observational studies. Bone. (2018) 116:58–66. doi: 10.1016/j.bone.2018.07.013, PMID: 30021126

[52] Dagdelen S Sener D Bayraktar M. Influence of type 2 diabetes mellitus on bone mineral density response to bisphosphonates in late postmenopausal osteoporosis. Adv Ther. (2007) 24:1314–20. doi: 10.1007/bf02877778, PMID: 18165214

[53] Zweifler LE Koh AJ Daignault-Newton S McCauley LK. Anabolic actions of PTH in murine models: two decades of insights. J Bone Mminer Res. (2021) 36:1979–98. doi: 10.1002/jbmr.4389, PMID: 34101904 PMC8596798

